# Can patient-reported profiles avoid unnecessary referral to a spine surgeon? An observational study to further develop the Nijmegen Decision Tool for Chronic Low Back Pain

**DOI:** 10.1371/journal.pone.0203518

**Published:** 2018-09-19

**Authors:** Miranda L. van Hooff, Johanna M. van Dongen, Veerle M. Coupé, Maarten Spruit, Raymond W. J. G. Ostelo, Marinus de Kleuver

**Affiliations:** 1 Department Research, Sint Maartenskliniek, Nijmegen, The Netherlands; 2 Department of Orthopaedic Surgery, Radboud University Medical Center, Nijmegen, The Netherlands; 3 Department of Health Sciences and the Amsterdam Public Health research institute, VU University, Amsterdam, The Netherlands; 4 Department of Epidemiology and Biostatistics, VU University Medical Center, Amsterdam, The Netherlands; 5 Department of Orthopedic Surgery, Sint Maartenskliniek, Nijmegen, The Netherlands; Medical College of Wisconsin, UNITED STATES

## Abstract

**Introduction:**

Chronic Low Back Pain (CLBP) is a heterogeneous condition with lack of diagnostic clarity. Therapeutic interventions show small effects. To improve outcomes by targeting interventions it is recommended to develop a triage system to surgical and non-surgical treatments based on treatment outcomes. The objective of the current study was to develop and internally validate prognostic models based on pre-treatment patient-reported profiles that identify patients who either respond or do not respond to two frequently performed treatments (lumbar spine surgery and multidisciplinary pain management program).

**Methods:**

A consecutive cohort study in a secondary referral spine center was performed. The study followed the recommendations of the PROGRESS framework and was registered in the Dutch Trial Register (NTR5946). Data of forty-seven potential pre-consultation (baseline) indicators predicting ‘response’ or ‘non-response’ at one-year follow-up for the two treatments were obtained to develop and validate four multivariable logistic regression models. The source population consisted of 3,410 referred CLBP-patients. Two treatment cohorts were defined: elective ‘spine surgery’ (n = 217 [6.4%]) and multidisciplinary bio-psychosocial ‘pain management program’ (n = 171 [5.0%]). Main inclusion criteria were age ≥18, CLBP (≥6 months), and not responding to primary care treatment. The primary outcome was functional ability: ‘response’ (Oswestry Disability Index [ODI] ≤22) and ‘non-response’ (ODI ≥41).

**Results:**

Baseline indicators predictive of treatment outcome were: degree of disability (all models), ≥2 previous spine surgeries, psychosocial complaints, age (onset <20 or >50), and patient expectations of treatment outcomes. The explained variances were low for the models predicting response and non-response to pain management program (*R*^2^ respectively 23% and 26%) and modest for surgery (*R*^2^ 30% and 39%). The overall performance was acceptable (c-index; 0.72–0.83), the model predicting non-response to surgery performed best (*R*^2^ = 39%; c-index = 0.83).

**Conclusion:**

This study was the first to identify different patient-reported profiles that predict response to different treatments for CLBP. The model predicting ‘non-response’ to elective lumbar spine surgery performed remarkably well, suggesting that referrals of these patients to a spine surgeon could be avoided. After external validation, the patient-reported profiles could potentially enhance timely patient triage to the right secondary care specialist and improve decision-making between clinican and patient. This could lead to improved treatment outcomes, which results in a more efficient use of healthcare resources.

## Introduction

Low back pain (LBP) causes the highest global burden of all diseases[1] by imposing a substantial economic burden on individuals, employers, the healthcare sector, and society as a whole due to increased work absenteeism, healthcare expenditures, and disability insurance[2,3].

Chronic LBP (CLBP; i.e. persistent LBP lasting over three months[4,5]) is a heterogeneous condition, which lacks diagnostic clarity. It is a common complaint for which patients seek consultation in primary care[6]. Although many primary care treatment options are available, it is estimated that 60–80% of the patients experience persistence of CLBP complaints after one year[7,8] and many patients consult secondary care spine specialists for their problems. However, secondary care providers cannot reliably identify which patients will benefit most from which surgical or a non‐surgical intervention, resulting in large practice variation [9–11]. Two frequently applied secondary care treatments are elective lumbar spine surgery and multidisciplinary bio-psychosocial pain management, e.g. combined physical and psychological (CPP) programs. In recent systematic reviews moderate quality evidence for small effects was found for both[10,11], but evidence is lacking on how to identify CLBP patients who are most likely to respond, or not, to these treatment options[9,11–13].

To improve decision-making, it has been recommended to develop a classification system (i.e. prognostic model)[14,15], based on patient profiles consisting of biomedical and psychosocial indicators that are thought to influence the outcomes of these interventions[12]. This system can be used for triaging CLBP patients to surgical and non-surgical secondary care specialists[9,11,13,16–19]. The American ‘National Institutes of Health’ (NIH) task force on research standards for CLBP recently supported this recommendation[20]. For this reason, the Nijmegen Decision Tool for CLBP (NDT-CLBP) has been developed[21] and implemented in the Dutch interface of SweSpine[22]. It consists of a web-based patient-reported screening questionnaire, in which indicators predicting treatment outcome are assessed, and includes systematic outcomes monitoring after treatment.

The purpose of this study is to develop and internally validate prognostic triage models for the NDT-CLBP, by identifying patient profiles, based on patient-reported indicators that either predict ‘response’ or ‘non-response’ to elective lumbar spine surgery, and ‘response’ or ‘non-response’ to a multidisciplinary bio-psychosocial treatment (i.e. an intensive CPP program).

## Materials and methods

### Study design

For the present observational study we used pre-consultation (baseline) and one-year follow-up data from a single institution spine outcome registry. Every CLBP patient referred to the outpatient orthopedic department routinely completes a web-based screening questionnaire before consultation. As part of routine outcomes monitoring, at one-year follow up after treatment, patients are asked to complete a web-based follow-up questionnaire, including various patient-reported outcome measures (PROMs). The screening questionnaire has been implemented in routine practice since May 2012 and data have been acquired systematically over time. The institution's internal review board approved the study. Ethical approval for this study was not required, as the Dutch Act on Medical Research involving Human Subjects does not apply to screening questionnaires that are part of routine clinical practice. Patient-data were obtained as a part of routine outcome monitoring for use in daily practice. All patients were informed on the procedure and had the opportunity to declare that (anonymized) data are not used for other purposes as scientific research. For this study, fully anonymized and de-identified data were obtained for analyses and report. During the course of the study, none of the researchers / authors had access to identifying information. We followed the recommendations of the PROGRESS framework[23] and of the TRIPOD statement[24]. The study is registered in the Dutch Trial Registry (NTR5946).

### Study population

**Fig 1:**
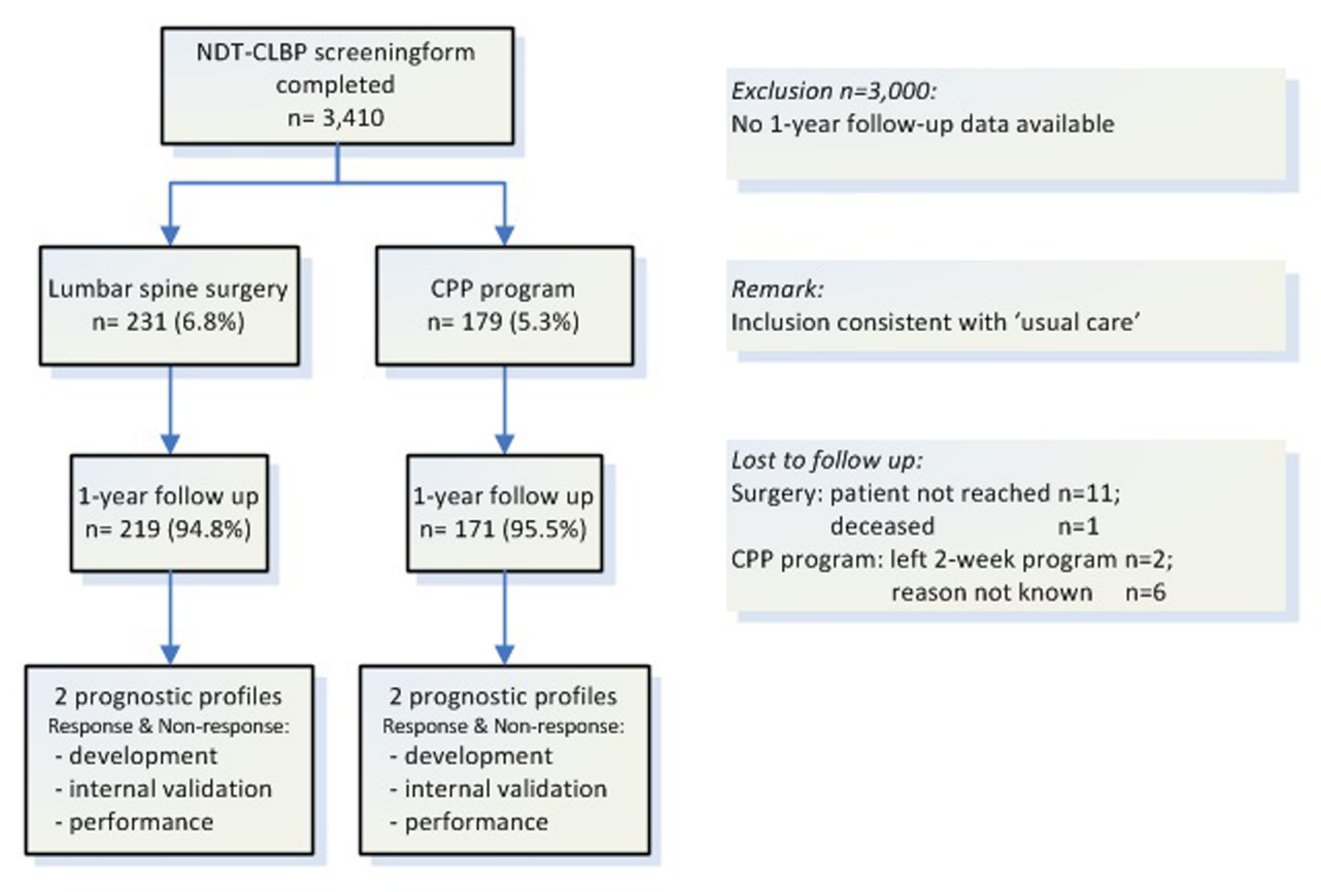
Flow of patient data throughout the study. *NDT-CLBP* Nijmegen Decision Tool for Chronic Low Back Pain. *CPP program* Combined Physical and Psychological program.

Data of patients who completed the questionnaire before consultation (baseline) between May 2012 and June 2014 were included, and one-year follow-up outcome data of treated patients were obtained. Treatment decision was based on standard care protocols, including patient history, the biomedical diagnostic phase (e.g. physical examination and imaging) and shared decision-making. From this source population, two distinct treatment cohorts were identified and included in the present study (Fig 1).

#### Lumbar spine surgery cohort

This cohort consisted of patients who underwent elective lumbar spine surgery (instrumented fusion with or without decompression). Complete baseline and follow-up data, were available for 219 patients (missing one-year follow up; n = 12 [5.2%]). Main inclusion criteria: age ≥18 years, CLBP (≥6 months) arising from degenerative lumbar spine disorders (including spinal stenosis, degenerative or isthmic spondylolisthesis or degenerative disc disease) confirmed by imaging, not responding to conservative treatment, with or without radiating leg pain.

#### Combined physical and psychological (CPP) program cohort

The CPP-program cohort comprised patients who participated in a multidisciplinary group-orientated 10-day residential program with a cognitive behavioral approach. The program included cognitive behavioral training, physical activities, and education[25]. Complete baseline and follow-up data were available for 171 patients (missing one-year follow up n = 8 [4.5%]). Main inclusion criteria: age ≥18, CLBP (≥6 months), not responding to primary care treatment, spine surgery is not an option, commitment to the program, which includes commitment to ‘stop shopping’, and willingness to change pain-related behavior.

### Outcome measure and definition of the outcome at follow up

The primary outcome was ‘response’ and ‘non-response’ in terms of functional ability at one-year follow up, as measured with the condition-specific Oswestry Disability Index (ODI, version 2.1a in Dutch)[26,27]. The total ODI score ranges from 0 to 100, with higher scores indicating greater disability[28]. ‘Response’ (yes/no; coded 1/0) was defined as having an ODI≤22 (i.e. successful, comparable to the ‘normal’ healthy population and an acceptable symptom state)[29,30]. ‘Non-response’ (yes/no; coded 1/0) was defined as having an ODI≥41 (i.e. failure, comparable to ‘severe disability’ and persistence of CLBP)[31]. *Note*: the definitions of ‘response’ and ‘non-response’ are not complementary (i.e. a ‘responder’ is not the opposite of a ‘non-responder’).

### Potential prognostic indicators

Forty-seven potential indicators, predicting treatment outcome or persistence of CLBP complaints, have been identified in a previous study and were included in the screening questionnaire of the spine registry (i.e. part of the NDT-CLBP)[21]. These indicators are classified into five domains (sociodemographic [n = 16], pain [n = 7], somatic [n = 14], psychologic [n = 8], and functioning & quality of life [n = 2]; see for more details S1 Table).

## Statistical analysis

### General

Patient characteristics were descriptively summarized. Complete-case analyses were deemed appropriate as less than 5% of the patients had missing values on one or more items. Before constructing and validating the final prediction models, the models’ assumptions were checked[32].

### Model development

To identify the prognostic indicators for ‘response’ and ‘non-response’, two-stage parsimonious backward logistic regression models were used. As all potential prognostic indicators were evidence-based and deemed clinically relevant (indicators mentioned in S1 Table), first statistical data reduction methods were applied. A univariate logistic regression analysis was performed and indicators with p≤0.10 were selected and subsequently included in an explorative oblique rotation principle component analysis (PCA)[33]. PCA was applied (1) to reduce the number of indicators and (2) to detect structure in the relationships between indicators, which is to classify indicators[32]. Second, the indicators identified in PCA with a factor loading ≥0.70 were included in the multivariable logistic regression analyses[32] (S2 Table). ‘BMI’ and ‘smoking’ were added in the multivariable analyses for surgery, because of the available evidence that high BMI[34–36] and smoking habit[35,37–41] are predictive for poor surgical outcome. Four multivariable logistic regression models were developed using a stepwise backward elimination method (stopping rule p<0.157, i.e. Akaike ‘s information criterion), and subsequently internally validated and checked for their performance[14,15,32,42,43]. In each regression model, the outcomes ‘response’ (successful outcome) and ‘non-response’ (failure) were the dependent variables and the predictive indicators were the independent variables.

The models’ performance was assessed by the percentage of variance explained (i.e. Nagelkerke’s *R*^2^); the agreement between the predicted probabilities of the outcome and the observed probabilities in the original data (p>0.05; i.e. Hosmer-Lemeshow test [HL-test]); and by the models’ discriminative ability (c-index: the area under the receiver operating characteristic curve [AUC])[32,43,44]. The c-index ranges from 0.5 (i.e. random prediction) to 1.0 (i.e. perfect prediction), where c-index >0.7 indicates that the model is acceptable[32].

An internal validation method with a bootstrap procedure (500 samples) was used to estimate the amount of over-fit. A slope value was calculated (i.e. closer to 1.0, less over-optimism)[43,44], and used to correct and shrink the regression coefficients, the *R*^*2*^, and the c-index[15,32,42,44].

Except for the model’s internal validation, performed in R (version 3.2.2. for Windows) and the scatter plots, created in STATA (version 12.0 for Windows; StataCorp, College Station, Texas, USA), statistical analyses were conducted in SPSS (version 22.0; IBM Corp, Amonk, New York, USA).

## Results

### Population at baseline (Tables 1 and S3 for complete overview)

**Table 1:** Main baseline patient characteristics.

		Source population	Lumbar spine surgery	CPP program
Domain	Characteristics	All (n = 3,410)	All (n = 219)	All (n = 171)
Sociodemographic	Age (years) [mean (SD) min-max]	50.8 (14.8) 18–84	53.6 (14.2) 15–83	45.1 (11.1) 20–71
	Gender Female [n (%)]	1,981 (58.1)	143 (65.3)	106 (62.0)
	Previous lumbar spine surgery—(Yes) [n(%)]	1,162 (34.1)	96 (43.8)	63 (36.8)
	Employed (Yes) [n(%)]	2,251 (66.0)	107 (48.9)	95 (55.6)
Pain	Duration—Back pain [n(%)]			
	3–12 months	729 (21.3)	44 (20.1)	16 (9.4)
	1–2 years	498 (14.6)	29 (13.2)	21 (12.3)
	> 2 years	2,183 (64.0)	146 (66.7)	134 (78.4)
	Duration Back pain >2 yrs [n(%)]			
	No	662 (19.4)	41 (18.7)	16 (9.4)
	2–10 years	1,523 (45.2)	107 (48.8)	83 (48.6)
	> 10 years	1,205 (35.3)	71 (32.4)	72 (42.1)
	NRS Back pain intensity [mean (SD) min-max]	7.0 (2.0) 0–10	6.9 (1.2) 0–10	7.6 (1.2) 3–10
	NRS Leg pain intensity [mean (SD) min-max]	5.4 (3.2) 0–10	6.1 (2.9) 0–10	5.4 (2.9) 0–10
Somatic	Co-morbidities—None [n(%)]	2,424 (71.1)	166 (75.8)	127 (74.3)
	≥ 1 Red Flag (Yes) [n(%)]	3,146 (92.3)	203 (92.7)	155 (90.6)
Psychological	SBT [n(%)]			
	Low risk	1,486 (32.6)	40 (18.3)	25 (14.6)
	Moderate risk	814 (23.9)	105 (47.9)	86 (50.3)
	High risk	1,110 (32.6)	74 (33.8)	60 (35.1)
	Expectations–recovery (Yes) [n(%)]	2,031 (59.6)	147 (67.1)	94 (55.0)
Functioning & Quality of life	ODI [mean (SD) min-max]	42.7 (16.1) 0–98	43.5 (15.5) 6–90	45.1 (13.4) 12–88
	SF36 PCS [mean (SD) min-max]	28.9 (8.3) 4–64	28.6 (7.5) 13–50	28.5 (6.1) 9–43
	SF6D [mean (SD) min-max]	0.576 (0.094) 0.294–0.921	0.572 (0.099) 0.296–0.892	0.561 (0.080) 0.366–0.843
	EQ5D [mean (SD) min-max]	0.447 (0.298) -0.329–1.000	0.444 (0.282) -0.204–0.893	0.405 (0.284) -0.134–0.843

*ODI* Oswestry Disability Index (version 2.1a in Dutch); *SBT* STarT Back Screening Tool (Dutch); *SF6D* Short Form 6 Dimensions; *SF36 PCS* Short Form 36 –Physical Component Scale; *EQ5D* EuroQol 5 Dimensions

The source population consisted of 3,410 referred patients who all completed the screening questionnaire and who consulted a spine surgeon. A total of 390 CLBP patients were included (mean age 49.9 [SD 13.6] years; 63.8% women; mean ODI 44.2 [SD 14.6]) and had lumbar spine surgery (n = 219, 6.4% of total population) or entered the CPP program (n = 171, 5.0% of total population).

### Functional outcomes at one-year follow up (Tables 1 and 2; Fig 2A and 2B)

The mean ODI of the surgery cohort improved from 43.5 (SD 15.5) at baseline to 30.4 (SD 18.7) at one-year follow up, and the mean ODI of the CPP-program cohort improved from 45.1 (SD 13.5) to 22.7 (SD 17.5) (Tables 1 and 2).

**Table 2:** Patient-reported outcomes at one year follow up.

	Lumbar spine surgery	CPP program
PROMs	All n = 219	All n = 171
NRS Back pain intensity [mean (SD) min-max]	4.4 (4.1) 0–10	4.2 (2.4) 0–9
ODI [mean (SD) min-max]	30.4 (18.7) 0–84	22.7 (17.5) 0–64
SF-36 PCS [mean (SD) min-max]	35.9 (10.0) 13–60	61.4 (20.5) 19–77
SF-36 MCS [mean (SD) min-max]	45.9 (10.9) 13–64	72.8 (18.1) 16–79
SF6D [mean (SD) min-max]	0.648 (0.130) 0.662–1.000	0.696 (0.125) 0.381–0.948
'response'—ODI ≤22 [n(%)]	82 (37.4)	86 (50.3)
'non-response'—ODI ≥41 [n(%)]	62 (28.3)	30 (17.5)

*PROMs* Patient-reported outcome measures *NRS* Numeric Rating Scale *ODI* Oswestry Disability Index (version 2.1a in Dutch) *SF36 PCS* Short Form 36—Physical Component Scale *SF36 MCS* Short Form 36—Mental Component Scale *SF6D* Short Form 6 Dimensions

**Fig 2:**
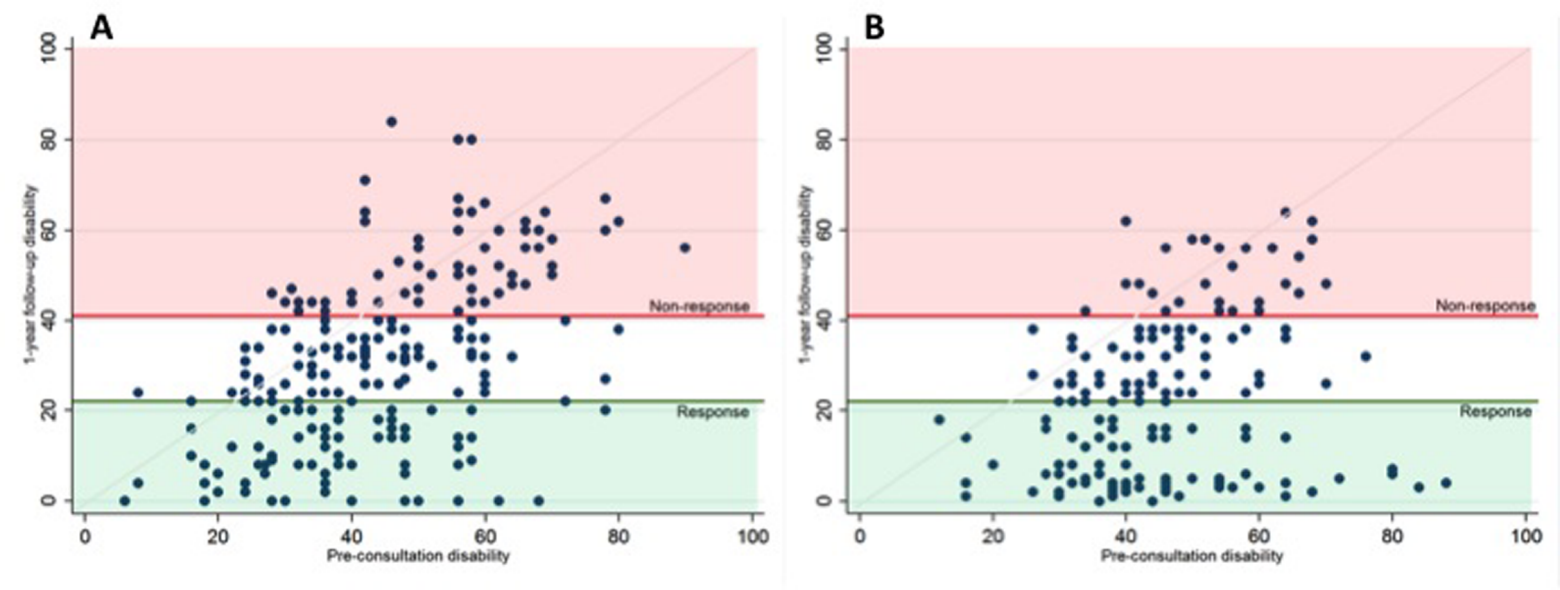
Functional status (ODI). **(A)** *Lumbar spine surgery cohort (n = 219)* and **(B)** *CPP-program cohort (n = 171)*. *ODI* Oswestry Disability Index; *disability* is measured with the ODI. The *x-axis* represents pre-consultation disability and the *y-axis* the one-year post-treatment disability. The *red area* above the *red horizontal line* represents ‘non-response’ (ODI≥41) and the *green area* under the *green horizontal line* represents ‘response’ (ODI≤22).

### Self-reported patient profiles

In Table 3 the final prognostic models and their performances are shown. For all four models the data fit well (HL-tests not significant). The final models showed low explained variances (*R*^*2*^), but overall acceptable performances (c-index). The slope values for the surgical ‘response’ and ‘non-response’ models showed less over-optimism then those for the CPP program (respectively 0.89 and 0.85 versus 0.79 and 0.75).

**Table 3:** Multivariable ‘response’ and ‘non-response’ models for lumbar spine surgery and combined physical and psychological (CPP) program.

	Spine Surgery								CPP program							
	Response: ODI ≤22				Non-response: ODI ≥41				Response: ODI ≤22				Non-response: ODI ≥41			
	B (SE)	OR	95% CI	p-value	B (SE)	OR	95% CI	p-value	B (SE)	OR	95% CI	p-value	B (SE)	OR	95% CI	p-value
ODI–screening	-0.04 (0.01)	0.96	0.94–0.98	<0.001	0.09 (0.02)	1.09	1.05–1.13	<0.001	-0.04 (0.01)	0.97	0.94–0.99	0.01	0.07 (0.02)	1.07	1.03–1.11	<0.001
Previous lumbar spine surgery																
n = 1 n ≥ 2 *reference*: *n = 0*	-0.49 (0.39) -1.67 (0.52)	0.62 0.19	0.29–1.32 0.07–0.52	0.21 0.001	0.32 (0.45) 1.49 (0.47)	1.41 4.25	0.57–3.51 1.68–10.75	0.46 0.001								
Employed	0.73 (0.33)	2.08	1.08–3.98	0.03												
STarT Back–screening																
Moderate risk High risk *reference*: *Low risk*					-1.66 (0.60) -1.08 (0.65)	0.17 0.29	0.05–0.58 0.08–1.10	0.01 0.07								
Somatisation																
Disagree Agree Strongly agree *reference*: *Strongly disagree*									0.88 (0.53) 0.32 (0.44) 1.30 (0.64)	2.42 0.73 3.67	0.86–6.83 0.31–1.72 1.04–12.94	0.10 0.47 0.04	0.20 (0.86) 1.25 (0.66) 1.51 (1.11)	0.82 3.48 0.22	0.15–4.45 0.96–12.65 0.03–1.95	0.82 0.06 0.17
Distress																
Yes, a little Yes, extremely *reference*: *No*									0.52 (0.38) -1.31 (0.64)	1.68 0.27	0.79–3.56 0.08–0.95	0.18 0.04	0.36 (0.55) 1.26 (0.02)	0.70 3.54	0.24–2.04 0.96–12.97	0.51 0.06
Expectations—recovery	1.17 (0.37)	3.22	1.57–6.69	0.001												
Red Flag																
Age (onset <20 or >50) Deformities	0.88 (0.33)	2.41	1.25–4.64	0.01	-1.16 (0.39) 0.84 (0.43)	2.41 2.61	1.25–4.64 1.09–6.23	0.01 0.03								
Neurological function																
Reduction muscle strength					0.92 (0.39)	2.50	1.16–5.36	0.02								
Paresthesia									0.85 (0.37)	0.43	0.21–0.88	0.02				
*Intercept*	0.05 (0.61)				-3.89 (0.68)				1.91 (0.75)				-5.59 (1.16)			
*Model performance*			*Model*	*Corrected*			*Model*	*Corrected*			*Model*	*Corrected*			*Model*	*Corrected*
Explained variance (Nagelkerke *R*^2^)			0.33	0.30			0.43	0.39			0.26	0.23			0.32	0.26
c-index			0.80	0.77			0.85	0.83			0.76	0.72			0.81	0.77
*Calibration*																
Hosmer and Lemeshow			Χ^2^ (8) = 3.51	p = 0.90			Χ^2^ (8) = 11.04	p = 0.20			Χ^2^ (8) = 6.09	p = 0.64			Χ^2^ (8) = 8.92	p = 0.35
Slope value			0.89				0.85				0.79				0.75	

*ODI* Oswestry Disability Index (version 2.1a in Dutch); *OR* Odds Ratio; *CI* Confidence Interval

Lumbar spine surgery
  Model predicting ‘response’ (c-index = 0.77; *R*^*2*^ = 30%): less functionally disabled (i.e. lower ODI), being employed, positive outcome expectations, and a Red Flag for age (i.e. age onset <20 or >50).
  Model predicting ‘non-response’ (c-index = 0.83; *R*^*2*^ = 39%): more functionally disabled (i.e. higher ODI), ≥2 previous spine surgeries, a moderate or high risk on STarT Back screening tool, a Red Flag for age (i.e. age onset <20 or >50), self-reported spinal deformity, and self-reported reduced muscle strength.
CPP program
  Model predicting ‘response’ (c-index = 0.72; *R*^*2*^ *=* 23%): less functionally disabled (i.e. low ODI), strong self-reported agreement with somatization, a little distressed, and self-reported paresthesia in the legs.
  Model predicting ‘non-response’ (c-index = 0.77; *R*^*2*^ = 26%): more functionally disabled (i.e. high ODI), self-reported agreement with somatization and extreme distress.

## Discussion

We present a tool to support triage of CLBP patients, based on their patient-reported pre-consultation profiles, to surgery and to a conservative treatment option (i.e. Nijmegen Decision Tool for Chronic Low Back Pain [NDT-CLBP]). To our knowledge, this is the first study to report such a tool, the development of which has been previously recommended[9,11–13]. We identified and internally validated pre-consultation patient-reported profiles that are predictive for either ‘response’ or ‘non-response’ to elective lumbar spine surgery and to a multidisciplinary bio-psychosocial treatment (a combined physical and psychological [CPP] program). Results indicate that different subgroups of patients could be identified in the heterogeneous CLBP population, without regard for the ‘anatomic’ of etiopathogenic diagnosis.

A remarkable finding is the good performance of the ‘non-response’ model for spinal surgery (*R*^*2*^ = 39%; c-index = 0.83; slope value 0.85). This indicates that the profile, based on patient-reported characteristics, seems accurate in identifying patients who are likely not to respond to surgical intervention. Within the source population (n = 3,410) of this study, 4.3% fulfil this patient-reported profile. If this profile remains stable after external validation, future CLBP patients who might not respond to surgery could be identified relatively well in an early phase, even before the patient consults the medical specialist. This could potentially change current practice; based on the profile, these patients could be triaged to a non-surgical medical spine specialist.

**Fig 3:**
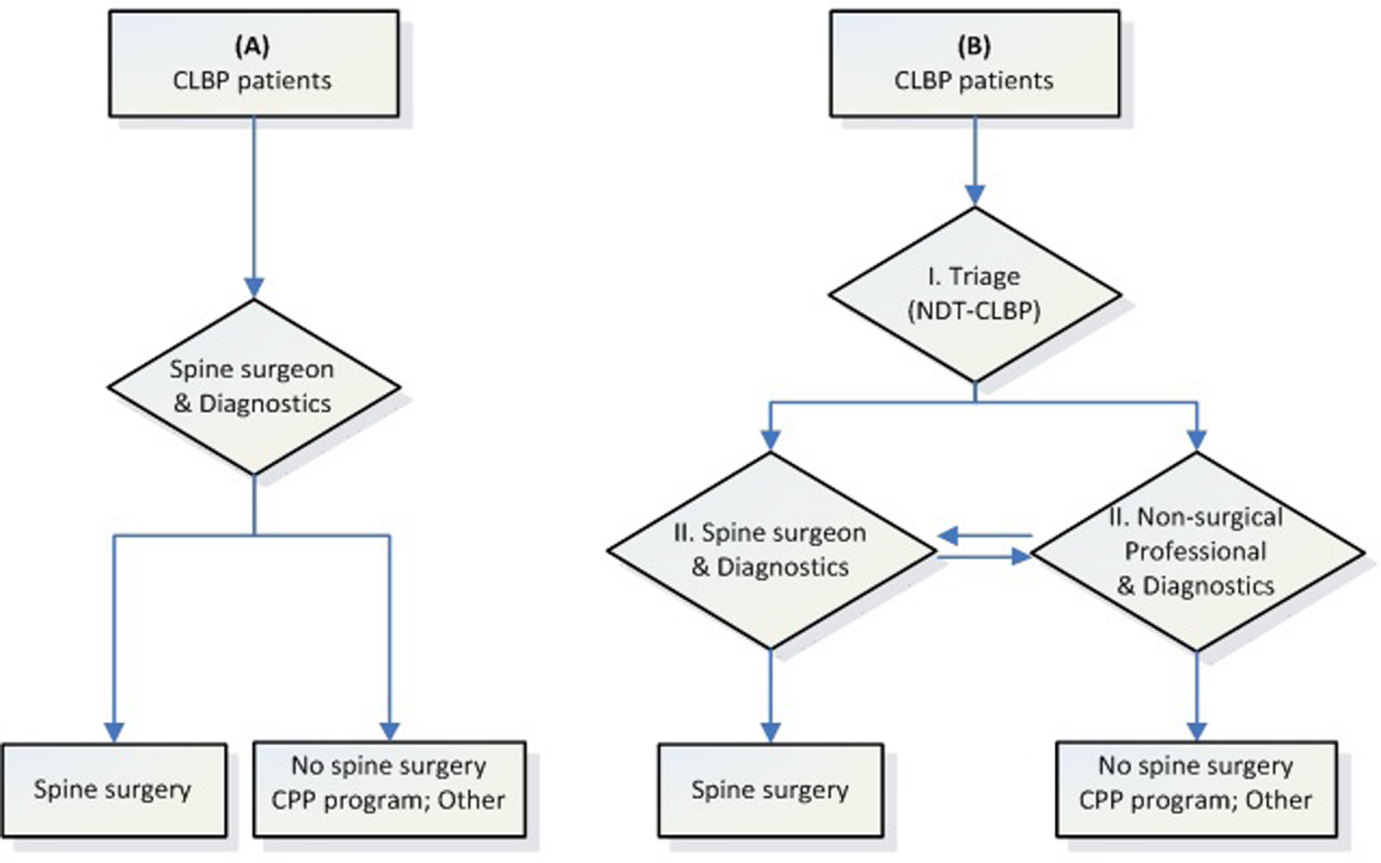
**The process of patient triage without (A) and with (B) the Nijmegen Decision Tool for Chronic Low Back Pain (NDT-CLBP; a two-staged decision process to guide diagnostics and treatment).** *CPP program* Combined Physical and Psychological program.

Overall, the final models’ performance was acceptable (all c-indexes satisfactory [>0.7]) and the explained variances of the surgical profiles were modest (*R*^*2*^ = 30–39%). However, the explained variances for the CPP-program models were rather low (*R*^*2*^ = 23–26%), which means that the predictive indicators only explain a fraction of the variance in outcome between patients. This was expected as only patient-reported data were used to develop the prognostic models and previous studies evaluating single treatment prediction models, have reported similar explained variances (*R*^*2*^ = 9% to 42%)[45–50]. Other indicators that are known to play a role in determining the outcome, which we intend to identify in the subsequent diagnostic phase, were not included in the models (e.g. specific diagnosis [e.g. degenerative spondylolisthesis, spinal stenosis] and clinical phenotypes, related to imaging [Modic changes] and biochemistry [biomarkers and genotypes])[51]. Future research should examine whether these indicators can extend and improve the current models, in order to have a reliable two-phased decision tool (1. triage to specialist and 2. based on diagnostics selection for treatment; Fig 3) with a correct combination of biomedical and psychosocial indicators. Then, stratification and personalized spine care might be achieved.

### Comparison with previous literature

Although several single treatment prognostic models for CLBP have been developed predicting the outcome of lumbar spine surgery[35,41,45,46,49,52–58] or multidisciplinary bio-psychosocial programs[47,48,50,59,60], we found no studies on prognostic models for different treatment options, which makes this study unique. Some of the studies found used predictive indicators derived from pre-treatment questionnaires[46–48] but the questionnaires and the outcome definitions used differed, which hamper comparison with the current study. The consistent finding that pre-treatment level of functioning (ODI) is predictive for the outcome in all the models is consistent with previous studies in spine surgery[41,46,49,56] and multidisciplinary bio-psychosocial programs[47,48,60]. However, other predictive indicators identified in these studies varied depending on the purpose of the study, and with that, on the choice of potential predictive factors derived from screening questionnaires, diagnostic phase and/or the intervention.

Although the outcomes ‘response’ and ‘non-response’ are defined differently in literature, consistent with previous studies important contributing factors for the surgical models were age[35,41,45,46,54,56,58], being employed[56,61], outcome expectancy[49,62–64], and previous lumbar spine surgery[41,54,56,58]. In the current study age <20 and >50 years was defined as a ‘red flag’ and was used as a screening question among other red flag screening questions (e.g. trauma; S1 Table). A recent paper by Premkumar et al. the authors confirmed an association between a positive answer to recent trauma and age >50 years and vertebral fracture as clinical (red flag) diagnosis [65], supporting the use of age and trauma as screening questions for patient triage to a surgeon for further diagnostics. Recently, Zehnder et al. found that an increasing number of previous surgeries were significantly associated with a less favorable outcome one year postoperatively. We found that two or more previous spine surgeries contribute substantially to a high risk of non-response to spine surgery. Patients with a history of prior spinal surgery should be critically evaluated before undergoing further spinal surgical interventions and the benefits of surgery should be carefully weighed up against the risks, since the outcome is predicted to be less favorable than for first-time procedures[58]. To avoid future often expensive revision surgery the NDT-CLBP seems appropriate to early identify patients ‘at risk’ and could contribute to ‘getting it right first time’ [66].

Growing evidence indicates that high BMI[35,36,41,56,58,67] and smoking[35,37–41,41,45,46,56,58,61] are predictive of a poor surgical outcome. However, we found no predictive value in the surgical non-response model for these indicators. A possible explanation could be that high BMI and smoking are predictive for post-operative complications, but might not diminish the final functional outcome. It could also be that self-reported BMI and smoking, as used in the current study, are less accurate than the use of objective measures[68–71], which would result in underestimation of their contribution to poor outcome and non-response in these cohorts.

Consistent with previous studies, predominantly psychological indicators were found to be predictive of the outcome of the CPP program, irrespective of the outcome definitions used[47,50], but there is a lack of high quality studies addressing this topic[50].

### Remarks on our study: Strengths and weaknesses

#### Strengths

An important strength of this study is that the NDT-CLBP is a first attempt to address a frequently recommended research theme: development of a classification system that is able to predict which CLBP patients benefit most from surgical or non‐surgical interventions[9,11–13]. Furthermore, the potential predictive indicators used to derive the final prognostic models were based on scientific evidence and multidisciplinary consensus[21] and comply with the recommendation to use a standard set of metrics for outcome reporting that matter to patients[72]. Patient outcome data and data of the potential predictive indicators were prospectively and systematically collected in a large web-based spine outcome registry, and a high response rate on the primary outcome measure (95%) at one-year follow up was achieved. We developed and internally validated parsimonious prognostic models, based on clinical relevance and corrected the models and the performance measures for over-optimism. To develop the prognostic models, we used the ODI as a single primary outcome to define ‘response’ and ‘non-response’ using evidence-based cut-off values. The application of these two distinct and strict absolute thresholds as outcome criteria is unique. In previous studies various measures of change on the ODI were used as an outcome, e.g. percentage improvement relative to the ODI baseline value[60] or reaching a threshold for minimal important change to indicate treatment success[46,73] or to indicate deterioration[41]. However, in the use of change measures methodological issues such as baseline-dependency are encountered[74]. This means that patients with severe disability could improve for example 10 points[75] or 16 points[60] on the ODI, whilst in fact they are still functionally disabled. In decision-making for interventions we think strict absolute thresholds are needed for clinical interpretations as ‘normal’ or ‘healthy’ state (‘response’) or persistence of CLBP complaints (‘non-response’).

#### Weaknesses

This study was performed in a single institution, which might result in limited generalizability to other secondary or tertiary settings treating CLBP patients. In line with the phases of prognostic research[42,43,76,77], future studies are planned to test the generalizability of the prognostic patient-reported profiles[14,43,44], to explore the interaction of these profiles between treatments, and to analyze their budget-impact and cost-effectiveness. Second, as recovery of functioning is a main goal in clinical practice we defined functional ability as the primary patient-related outcome domain of surgical and non-surgical interventions. However, other outcome domains might also be relevant in spine care (i.e. pain, health-related quality of life[72,78–80], complications[72,80], repeat spine surgery, work status, and analgesic use[72]). Thirdly, patient-reported red flags are part of the screening questionnaire of the NDT-CLBP [21] and recommended in international guidelines [16–18]. The present study shows that most of the patients with CLBP (92%; Table 1) have at least one positive red flag but do not have a serious underlying condition. Taking the guideline recommendations literally could cause harm (e.g. unnecessary diagnostics, unnecessary exposure to radiation, unnecessary treatments, including surgery). In a recent paper by Premkumar et al. [65] the authors concluded that while a positive response to a red flag question might indicate the presence of disease, a negative response to 1 or 2 red flag questions does not meaningfully decrease the likelihood of a red flag diagnosis. We agree with the authors that caution should be taken against the use of red flags alone as a screening tool CLBP is characterized by a complex system of multiple interacting bio-psycho-social indicators. Using ‘red flags’ with binary yes/no outcomes, does no justice to this complexity, oversimplifies the problem, and may harm patients. For example, in a certain context and combination of influencing factors, age may be a red flag, and in another situation it may not. Furthermore, after screening using a triage tool, a clinical assessment will always be needed, which will provide significant further input for decision making. The screening tool itself will never come to a diagnosis. We expect that combinations of ‘flags’ and clinical features, determined in the diagnostic phase, might be more informative to assist in clinical decision-making. This will be further explored in the second phase of the NDT-CLBP (Fig 3) when patient profiles for decision to treatment, rather than triage alone, will be analyzed and built. Fourth, per prognostic model a relatively high number of potential predictive indicators (i.e. n = 47) were used compared to the number of events occurred (i.e. ‘response’ or ‘non-response’). Based on clinical relevance and supported by a data driven approach, only the most contributing prognostic indicators could be included in the multivariable logistic regression models for internal validation, which might lead to missing weaker prognostic factors. Moreover, ‘confounding by indication’ might have been introduced due to the observational design used. As the amount of potential predictive indicators was high, we were not able to add interaction terms to our models, which might have led to too optimistic (although we corrected for this optimism[14,15,32,42,43,81]) and less accurate models. Fifth, and related to the previous point, a classical multivariable logistic regression was chosen to develop and validate the models, as it produces relatively stable predictions and in relatively small datasets in particular[82], and limitations of this method for clinical practice are known. Bayesian network methodology could overcome these limitations as well as the previously mentioned weakness of the high amount of potential predictive indicators compared to the number of events occurred. The method allows for making predictions at various times during a health care process, each time using all the available information of the patient concerned[83]. However, it is currently unknown whether such models lead to better validity and more research is needed to determine the usefulness in clinical practice.

### Conclusion and implications for clinical practice

Based on pre-consultation patient-reported characteristics and treatment outcomes different prognostic profiles were identified that may contribute to triage of CLBP patients to the right surgical or non-surgical specialist for consultation. The ‘non-response’ profile for elective lumbar spine surgery performed remarkably well. If successful, based on their patient-reported profile, these patients could be triaged to a non-surgical specialist and unnecessary and unhelpful surgeries could be avoided. In future when the prognostic patient-reported profiles remain stable, the NDT-CLBP could enhance timely triage patient to the right clinician and contributes to decision-making between clinician and patient. The profiles support improvement of both surgical and conservative treatment outcomes that ultimately results in a more efficient use of healthcare resources.

## Supporting information

S1 Table Indicators and coding. (PDF)

S2 Table Selected indicators. (PDF)

S3 Table Complete overview of results per indicator. (PDF)

S1 Checklist Completed STROBE checklist cohort studies. (PDF)
