# Supplementary material for: Can patient-reported profiles avoid unnecessary referral to a spine surgeon? An observational study to further develop the Nijmegen Decision Tool for Chronic Low Back Pain
Source: PLoS One. 2018 Sep 19;13(9):e0203518. doi: 10.1371/journal.pone.0203518 (PMC6145570; doi:10.1371/journal.pone.0203518)
Supplement: S1 Table — (PDF) [file pone.0203518.s001.pdf]

S1 Table. Indicators and coding.

Domain	Category	Evidence	Measurement
Sociodemographic	*Personal*		
	Age	I	Years
		I	Categorized: ≤25; 26-50; 51-75; >75
	Gender	I	Female=0; Male=1
	Body Mass Index	I	Kg/ m2; Self-reported weight/height
	Health		
	Smoking	I	No=0; Yes=1
	Previous back surgery	C	No=0; Yes=1
			Number of surgeries categorized: 0; 1; ≥2
	Use of analgesics	I	No=0; Yes=1
	Self-management of complaints	C	XX
	Interventions in the past	C	Previous non-invasive treatment(s): No=0; Yes=1
	Social		
	Social status	I	Social status: Married; Living together; Single
	Social support	C	No=0; Yes=1
	Wotk		
	Socio-economic status	I X	Educational level: low=lower secondary education or less, medium=higher secondary education, high=college or university
		C	At work (employed): No=0; Yes=1
			Currently out of work (unemployed): No=0; Yes=1
		I	Paind sick leave: No; Yes fully, due to back problems; Yes partly, due to back problems; Yes, due to other disease
		I X	Physically demanding work: No=0; Yes=1
	Work satisfaction	I	Dissatisfied=0; Satisfied=1
	Functioning - work	I	At work despite back pain complaints: No=0; Yes=1
	Sick leave	C	Being on sick leave due to back complaints; No=0; Yes=1
	Litigation	C	No=0; Yes=1
Pain	Pain duration	I	Back pain duration: no back pain; <3 months; 3-12 months; 1-2 years; 2-5 years; 6-10 years; >10 years
			Duration radiating leg pain: no radiating leg pain; <3 months; 3-12 months; 1-2 years; >2 years
	Back pain intensity	C	Numeric rating scale (range: 0-10)
	Leg pain intensity	C	Numeric rating scale (range: 0-10)
	Preceding (prior) back pain episodes	I	Prior back pain episodes: No=0; Yes=1
			Number
	Daily course of pain complaints	C	Red flag Nightly pain: No=0; Yes=1
	Influence of rest, mobility, and posture	C	XX

Somatic	Diagnosis; co-morbidities (Red Flag)	C	Diagnosis: not reported *<br>Comorbidieties: none; heart problems; neurological problems; cancer; other diseases limiting walking capacitys; other disease causing pain
	Bulging or protruding disc	C	XX *
	Loss of neurological function	C	Incontinence urine/faeces (sphincter): No=0; Yes=1<br>Saddle anesthesia/numbness: No=0; Yes=1<br>Numbness leg/foot: No=0; Yes=1<br>Loss of muscle strenth leg/foot: No=0; Yes=1<br>Paesthesia leg/foot: No=0; Yes=1
	Red flags:	C	
	Pain started age <20 or >50 years		No=0; Yes=1
	Significant trauma		No=0; Yes=1
	Pain is constant and non-mechanical		No=0; Yes=1
	Pain in thoracic spine		No=0; Yes=1
	Deformities (e.g. scoliosis)		No=0; Yes=1
	Previous history of malignancies		No=0; Yes=1
	History of intravenous drug use		No=0; Yes=1
	AIDS/HIV		No=0; Yes=1
	Current steroid use		No=0; Yes=1
	Recent unexplained weight loss		No=0; Yes=1
	Nightly pain		No=0; Yes=1
	Inflammatory bowel disease		No=0; Yes=1
Psychological	*Psychic affect*		
	Distress	C	No; Yes a litte; Yes very much<br>SBT: Low risk; Moderate risk; High risk
	Anxiety	C	No; Yes a litte; Yes very much<br>SBT: Low risk; Moderate risk; High risk
	Cognition		
	Catastrophizing	C	SBT item 7: agree; disagree<br>SBT: Low risk; Moderate risk; High risk
	Somatization	C	My body is telling me I have something dangerously wrong: Strongly disagree; Disagree; Agree; Strongly agree<br>SBT: Low risk; Moderate risk; High risk
	Coping	C	SBT: Low risk; Moderate risk; High risk
	Behavior		
	Fear of movement/(re)injury	C	SBT item 5: agree; disagree<br>SBT: Low risk; Moderate risk; High risk
	Expectations – return to work	C	Do you expect to be able to return to work? Currently at work; Return to work (full-time); Return to work (part-time); Change jobs; Keep receiving disability pensions; Keep my retirement benefits/alimony
	Expectations – outcome interventions	C	(recovery) Do you expect to be free of complaints after treatment? No=0; Yes=1

Functioning & Quality of life	Functioning in daily activities & walking	C	Oswestry Disability Index (range: 0-100)
			How far are you able to walk? <100 meter; 100-500 meter; 500 meter - 1 kilometer; >1 kilometer
			Practice sports? No; Yes top level; Yes recreational level
	Pain-inference daily activities	C	Disability in leisure activities? No=0; Yes=1
			SBT item 9 Bothersomeness: No=0; Yes=1
	Health-related physical functioning	C	SF-36 – Physical Component Summary (range: 0-100)
			SF-36 – Mental Component Summary (range: 0-100)
			SF6D
		C	EQ5D
			EQ5D - Visual analogue scale (0-100)

C Conclusive evidence & consensus for predictive value; *I* Inconclusive evidence; *X* Consensus for no predictive value or overlap with other indicators [21]
* Not patient-reported; XX Not questioned
ODI Oswestry Disability Index (version 2.1a in Dutch); *SBT*, STarT Back Screening Tool (Dutch version); *SF36* Short form 36; *SF6D* Short Form 6 Dimensions; *EQ5D* EuroQol 5 Dimensions
