# Supplementary material for: Can patient-reported profiles avoid unnecessary referral to a spine surgeon? An observational study to further develop the Nijmegen Decision Tool for Chronic Low Back Pain
Source: PLoS One. 2018 Sep 19;13(9):e0203518. doi: 10.1371/journal.pone.0203518 (PMC6145570; doi:10.1371/journal.pone.0203518)
Supplement: S2 Table — (PDF) [file pone.0203518.s002.pdf]

S2 Table. Selected indicators.

Domain	Characteristics	Lumbar spine surgery		CPP program	
		**Response (ODI ≤22)**	**Non-Response (ODI≥41)**	**Response (ODI ≤22)**	**Non-Response (ODI≥41)**
Demographic	Gender				X
	Body Mass Index (kg/m2)		X		
	Smoking				
	Previous back surgery; number 0 : 1 : ≥2	X	X		
	Social support	X			
	Employed	X	X		
	Work satisfaction			X	
	Sick leave				X
Pain	Duration - Leg pain	X		X	
	NRS Leg pain intensity				
	Daily course - Nightly pain (RF)		X		
Somatic	Co-morbidities				X
	Loss of neurological function				
	Incontinence urine/faeces	X			
	Numbness leg/foot			X	
	Loss of muscle strength leg/foot		X		
	Paresthesia leg/foot		X	X	
	Red flags				
	Pain started age <20 or >50 years	X	X		
	Deformities (e.g. scoliosis)		X		
Psychological	SBT	X	X		
	Distress			X	X
	Anxiety				X
	Somatization			X	X
	Expectations – return to work	X	X	X	
	Expectations – recovery	X			
Functioning & Quality of life	ODI	X	X	X	X
	Walking distance			X	X
	SBT item 9 - Bothersomeness	X			
	SF-36 PCS	X		X	

CPP Combined Physical and Psychological; *RF* red flag; *ODI* Oswestry Disability Index (version 2.1a in Dutch*);* *SBT* STarT Back Screening Tool (Dutch version)*; SF36-PCS* Short Form 36 - Physical Component Scale
