# Supplementary material for: Can patient-reported profiles avoid unnecessary referral to a spine surgeon? An observational study to further develop the Nijmegen Decision Tool for Chronic Low Back Pain
Source: PLoS One. 2018 Sep 19;13(9):e0203518. doi: 10.1371/journal.pone.0203518 (PMC6145570; doi:10.1371/journal.pone.0203518)
Supplement: S3 Table — (PDF) [file pone.0203518.s003.pdf]

S3 Table. Complete overview of results per indicator.

Domain	Characteristics	Source population	Lumbar spine surgery			CPP program		
		**All (**n= 3,410)	**All** n= 219	**Response (ODI ≤22)** n= 82 (events)	**Non-Response (ODI≥41)** n= 62 (events)	**All** n= 171	**Response (ODI ≤22)** n= 86 (events)	**Non-Response (ODI≥41)** n= 30 (events)
Sociodemographic	*Personal*							
	Age (years) [mean (SD) min-max]	50.8 (14.8) 18-84	53.6 (14.2) 15-83	51.1 (14.5) 15-77	57 (11.1) 33-83	45.1 (11.1) 20-71	43.0 (11.3) 23-67	47.9 (10.4) 23-67
	≤25 : 26-5 : 51-75 : ≥76) [n(%)]	143 (4.2) : 2,157 (63.3) : 996 (29.2) : 116 (3.3)	9 (4.1) : 82 (37.4) : 119 (54.3) : 9 (4.1)	4 (4.9) : 35 (42.7) : 41 (50.0) : 2 (2.4)	0 : 19 (30.6) : 41 (66.1) : 2 (3.2)	7 (4.1) : 109 (63.7) : 55 (32.2) : 0	4 (4.7) : 59 (68.6) : 23 (26.7) : 0	1 (3.3) : 15 (50.0) : 14 (46.7) : 0
	Gender Female [n (%)]	1,981 (58.1)	143 (65.3)	48 (58.5)	43 (69.4)	106 (62.0)	52 (60.5)	14 (46.7)
	Body Mass Index (kg/m2) [mean (SD)]	29.4 (4.2)	26.0 (4.9)	25.7 (3.3)	26.9 (7.5)	26.6 (6.1)	26.4 (4.2)	28.2 (5.3)
	Health							
	Smoking (Yes) [n(%)]	935 (27.4)	70 (32.0)	27 (32.9)	23 (37.1)	55 (32.2)	32 (37.2)	6 (20.0)
	Previous back surgery							
	(Yes)) [n(%)]	1,162 (34.1)	96 (43.8)	22 (26.8)	38 (61.3)	63 (36.8)	29 (33.7)	13 (43.3)
	Number 0 : 1 : ≥2) [n(%)]	2,235 (65.5) : 650 (19.1) : 525 (15.4)	123 (56.2) : 53 (24.2) : 43 (19.4)	60 (73.2) : 16 (19.5) : 6 (7.3)	24 (38.7) : 16 (25.8) : 22 (35.5)	108 (63.2) : 38 (22.2) 25 (14.6)	57 (66.3) : 18 (20.9) : 11 (12.8)	17 (56.7) : 7 (23.3) : 6 (20.0)
	Use of analgesics (Yes) [n(%)]	2,179 (87.4)	195 (89)	70 (85.4)	59 (95.2)	160 (93.6)	79 (91.9)	28 (93.3)
	Previous non-invasive treatment(s) (Yes) [n(%)]	2,636 (77.3)	174 (79.5)	61 (74.4)	53 (85.50	140 (81.9)	72 (83.7)	25 (83.3)
	Social							
	Social status [n(%)]							
	Married	2,113 (62.0)	155 (70.8)	61 (74.4)	45 (72.6)	122 (71.3)	58 (67.4)	25 (83.3)
	Living together	462 (13.5)	19 (8.7)	9 (11.0)	3 (4.8)	27 (12.9)	14 (16.3)	5 (16.7)
	Single	835 (24.5)	45 (20.5)	12 (14.6)	14 (22.6)	22 (12.9)	14 (16.3)	0
	Social support (Yes) [n(%)]	1,929 (56.6)	133 (60.7)	37 (45.1)	45 (72.6)	114 (66.7)	55 (64.0)	22 (73.3)
	Work - Socio-economic status							
	Educational level [n (%)]							
	Low	1,809 (53.0)	111 (50.7)	37 (45.1)	38 (61.3)	72 (42.1)	31 (36.0)	18 (60.0)
	Intermediate	864 (25.4)	65 (29.7)	25 (30.5)	16 (25.8)	67 (39.2)	37 (43.0)	12 (40.0)
	High	737 (21.6)	43 (19.6)	20 (24.4)	8 (12.9)	32 (18.7)	18 (20.9)	0

	Employed (Yes) [n(%)]	2,251 (66.0)	107 (48.9)	52 (63.4)	23 (37.1)	95 (55.6)	52 (60.5)	15 (50.0)
	Currently unemployed (Yes) [n(%)]	857 (25.1)	50 (22.8)	11 (13.4)	18 (29.0)	54 (31.6)	24 (27.9)	10 (33.3)
	Paid sick leave [n(%)]							
	No	2,591 (76.0)	166 (75.8)	66 (80.5)	43 (69.4)	91 (53.2)	47 (54.7)	9 (30.0)
	Yes fully, due to back problems	517 (15.2)	34 (15.5)	8 (9.8)	13 (21.0)	58 (53.9)	30 (34.9)	14 (46.7)
	Yes partly, due to back problems	248 (7.3)	17 (7.8)	6 (7.3)	6 (9.7)	19 (11.1)	7 (8.1)	7 (23.3)
	Yes, due to other disease	54 (1.6)	2 (0.9)	2 (2.4)	0	3 (1.8)	2 (2.3)	0
	Physically demanding work [n(%)]							
	(Yes) [n(%)]	415 (12.2)	21 (9.6)	11 (13.4)	4 (6.5)	21 (12.8)	16 (18.6)	2 (6.7)
	No : Light : Medium : Heavy	1,414 (41.5) : 541 (15.9) : 1,040 (30.5) : 415 (12.2)	86 (39.3) : 34 (15.5) : 78 (35.6) : 21 (9.6)	23 (28.0) : 16 (19.5) : 32 (39.0) : 11 (13.4)	29 (46.8) : 11 (17.7) : 18 (29.0) : 4 (6.5)	60 (35.1) : 19 (11.1) : 71 (41.5) : 21 (12.3)	25 (29.1) : 9 (10.5) : 36 (41.9) : 16 (18.6)	10 (33.3) : 4 (13.3) : 14 (46.7) : 2 (6.7)
	Work satisfaction (Yes) [n(%)]	2,152 (63.1)	147 (67.1)	61 (74.4)	36 (58.1)	109 (63.7)	60 (69.8)	16 (53.3)
	Functioning - work - At work despite CLBP [n(%)]							
	No : Yes partly; Yes fully	1,460 (42.8) : 1,479 (43.4) : 471 (13.8)	87 (39.7) : 108 (49.3) : 24 (11.0)	22 (26.8) : 42 (51.2) : 18 (22.0)	34 (54.8) : 25 (40.3) : 3 (4.8)	95 (55.6) : 63 (36.8) : 13 (7.6)	44 (51.2) : 33 (38.4) : 9 (10.5)	22 (73.3) : 8 (27.7) : 0
	Sick leave (Yes) [n(%)]	822 (24.1)	51 (23.3)	14 (17.1)	19 (30.6)	78 (45.6)	37 (43.0)	21 (70.0)
	Litigation (Yes) [n(%)]	132 (3.9)	4 (1.8)	2 (2.4)	2 (3.2)	6 (3.5)	5 (5.8)	0
Pain	Duration - Back pain [n(%)]							
	No back pain	0	7 (3.2)	0	0	0	0	0
	< 3 months	0	4 (1.8)	0	0	0	0	0
	3-12 months	729 (21.3)	33 (15.1)	18 (22.0)	6 (9.7)	16 (9.4)	11 (12.8)	3 (10.0)
	1-2 years	498 (14.6)	29 (13.2)	7 (8.5)	12 (19.4)	21 (12.3)	11 (12.8)	5 (16.7)
	> 2 years	2,183 (64.0)	146 (66.7)	57 (69.5)	44 (71.0)	134 (78.4)	64 (74.4)	22 (73.3)
	Duration Back pain >2 yrs [n(%)]							
	No	662 (19.4)	41 (18.7)	18 (22.0)	8 (12.9)	16 (9.4)	10 (11.6)	3 (10.0)
	2-5 years	1,020 (29.9)	73 (33.3)	28 (34.1)	24 (38.7)	54 (31.6)	27 (31.4)	12 (40.0)
	6-10 years	523 (15.3)	34 (15.5)	11 (13.4)	11 (17.7)	29 (17.0)	13 (15.1)	5 (16.7)
	> 10 years	1,205 (35.3)	71 (32.4)	25 (30.5)	19 (30.6)	72 (42.1)	36 (41.9)	10 (33.3)
	Duration - Leg pain [n(%)]							
	No radiating leg pain	751 (22.0)	32 (16.6)	16 (19.5)	5 (8.1)	29 (17.0)	19 (22.1)	2 (6.7)
	< 3 months	188 (5.5)	9 (4.1)	3 (3.7)	1 (1.6)	8 (4.7)	5 (5.8)	1 (3.3)

	3-12 months	705 (20.7)	40 (18.3)	14 (17.1)	10 (16.1)	35 (20.5)	15 (17.4)	8 (26.7)
	1-2 years	548 (16.1)	38 (17.4)	15 (18.3)	11 (17.7)	34 (19.9)	17 (19.8)	6 (20.0)
	> 2 years	1,208 (35.7)	100 (45.7)	34 (41.5)	35 (56.5)	65 (38.0)	30 (34.9)	13 (43.3)
	NRS Back pain intensity [mean (SD) min-max]	7.0 (2.0) 0-10	6.9 (1.2) 0-10	6.5 (2.4 0-10	7.7 (1.8) 0-10	7.6 (1.2) 3-10	7.5 (1.2) 4-10	8.0 (1.1) 6-10
	NRS Leg pain intensity [mean (SD) min-max]	5.4 (3.2) 0-10	6.1 (2.9) 0-10	5.5 (3.0) 0-10	7.2 (2.6) 0-10	5.4 (2.9) 0-10	4.8 (3.0) 0-10	6.7 (2.4) 0-9
	Preceding back pain episodes [n(%)]	2,290 (67.4)	158 (72.1)	59 (72)	46 (74.2)	127 (74.3)	67 (77.9)	22 (73.3)
	Daily course - Nightly pain (RF; Yes) [n(%)]	1,729 (50.7)	101 (46.1)	34 (41.5)	36 (58.1)	98 (57.3)	47 (54.7)	17 (56.7)
Somatic	Co-morbidities [n(%)]							
	None	2,424 (71.1)	166 (75.8)	67 (81.7)	38 (61.3)	127 (74.3)	64 (74.4)	19 (63.3)
	Heart problems	92 (2.7)	5 (2.3)	1 (1.2)	3 (4.8)	2 (1.2)	1 (1.2)	1 (3.3)
	Neurological problems	247 (7.2)	19 (8.7)	7 (8.5)	7 (11.3)	15 (8.8)	6 (7.0)	3 (10.0)
	Cancer	18 (0.5)	1 (0.5)	0	1 (1.6)	0	0	0
	Other diseases limiting walking capacity	255 (7.5)	14 (6.4)	4 (4.9)	7 (11.3)	9 (5.3)	2 (2.3)	5 (16.7)
	Other diseases causing pain	374 (11.0)	16 (6.4)	3 (3.7)	6 (9.7)	18 (10.5)	13 (15.1)	2 (6.7)
	Loss of neurological function (Yes) [n(%)							
	≥ 1 loss in neurological function	2,679 (78.6)	182 (83.1)	64 (78)	57 (91.9)	144 (84.2)	70 (81.4)	28 (93.3)
	Incontinence urine/faeces	411 (12.1)	31 (14.2)	6 (7.3)	16 (25.8)	21 (12.3)	11 (12.8)	5 (16.7)
	Saddle anestesia	565 (16.6)	37 (16.9)	11 (13.4)	14 (22.6)	31 (18.1)	15 (17.4)	8 (26.7)
	Numbness leg/foot	1,213 (35.6)	93 (42.5)	28 (34.1)	38 (61.3)	66 (38.6)	28 (17.4)	12 (40.0)
	Loss of muscle strength leg/foot	1,778 (52.1)	115 (52.2)	39 (47.6)	44 (71.0)	83 (48.5)	43 (50.0)	19 (63.3)
	Paresthesia leg/foot	2,006 (58.8)	141 (64.4)	50 (61.0)	47 (75.8)	113 (66.1)	48 (55.8)	21 (70.0)
	Red flags (Yes) [n(%)]							
	≥ 1 Red flag	3,146 (92.3)	203 (92.7)	75 (91.5)	60 (96.8)	155 (90.6)	77 (89.5)	27 (90.0)
	Pain started age <20 or >50 years	1,703 (49.9)	108 (49.3)	47 (57.3)	25 (40.3)	87 (50.9)	44 (51.2)	14 (46.7)
	Significant trauma	567 (16.6)	37 (16.9)	15 (18.3)	15 (24.2)	29 (17.0)	12 (14.0)	8 (26.7)
	Pain is constant and non-mechanical	2,475 (72.6)	164 (74.9)	54 (65.9)	54 (87.1)	131 (76.6)	62 (72.1)	25 (83.3)
	Pain in thoracic spine	315 (9.2)	13 (5.9)	3 (3.7)	2 (3.2)	15 (8.8)	9 (10.5)	1 (3.3)
	Deformities (e.g. scoliosis)	728 (21.3)	45 (20.5)	13 (15.9)	18 (29.0)	35 (20.5)	21 (24.4)	3 (10.0)
	Previous history of malignancies	131 (3.8)	9 (4.1)	3 (3.7)	3 (4.8)	5 (2.9)	3 (3.5)	0

	Current steroid use	126 (3.7)	7 (3.2)	2 (2.4)	4 (6.5)	9 (5.3)	3 (3.5)	4 (13.3)
	History of intravenous drug use	8 (0.2)	0	0	0	0	0	0
	AIDS/HIV illness	1 (0.1)	0	0	0	0	0	0
	Psoriasis	142 (4.2)	3 (1.4)	1 (1.2)	2 (3.2)	2 (1.2)	0	2 (6.7)
	Recent unexplained weight loss	113 (3.3)	8 (3.7)	2 (2.4)	3 (4.8)	8 (4.7)	5 (5.8)	2 (6.7)
	Inflammatory bowel disease	31 (0.9)	3 (1.4)	1 (1.2)	1 (1.6)	0	0	0
	≥ 1 Red flag or loss in neuological function	3,312 (97.1)	211 (96.3)	78 (85.1)	61 (98.4)	211 (96.3)	82 (96.3)	29 (96.7)
Psychological	SBT [n(%)]							
	Low risk	1,486 (32.6)	40 (18.3)	23 (28.0)	9 (14.5)	25 (14.6)	17 (19.8)	1 (3.3)
	Moderate risk	814 (23.9)	105 (47.9)	42 (51.2)	22 (35.5)	86 (50.3)	46 (53.3)	12 (40.0)
	High risk	1,110 (32.6)	74 (33.8)	17 (20.7)	19 (30.6)	60 (35.1)	23 (26.7)	17 (56.7)
	Distress [n(%)]							
	No; Yes a little; Yes very much	1,822 (53.4) : 1,291 (37.9) : 297 (8.7)	119 (54.3) : 78 (35.6) : 22 (10.0)	51 (62.2) : 25 (30.5) : 6 (7.3)	30 (48.4) : 24 (38.7) : 8 (12.9)	80 (46.8) : 67 (39.2) : 24 (14.0)	42 (48.9) : 39 (45.3) : 5 (5.8)	10 (33.3) : 10 (33.3) : (33.3)
	Anxiety [n(%)]							
	No; Yes a little; Yes very much	2,031 (59.6) : 1,070 (31.4) : 309 (9.1)	125 (57.1) : 75 (34.2) : 19 (8.7)	50 (61.0) : 28 (34.1) : 4 (4.9)	30 (48.4) : 20 (32.3) : 12 (19.4)	94 (55.0) : 60 (35.1) : 17 (9.9)	50 (58.1) : 30 (34.9) : 6 (7.0)	11 (36.7) : 15 (50.0) : 4 (13.3)
	Somatization [n(%)]							
	Strongly disagree	667 (19.6)	34 (15.5)	15 (18.3)	9 (14.5)	36 (21.1)	17 (19.8)	4 (13.3)
	Disagree	737 (21.6)	49 (22.4)	18 (22.0)	14 (22.6)	34 (19.9)	23 (26.7)	3 (10.0)
	Agree	1,510 (44.3)	104 (47.5)	38 (46.3)	27 (43.5)	75 (43.9)	29 (33.7)	21 (70.0)
	Strongly agree	496 (14.5)	32 (14.6)	11 (13.4)	12 (19.4)	26 (15.2)	17 (19.8)	2 (6.7)
	Expectations – return to work [n(%)]							
	Currently at work	1,160 (34.6)	69 (31.5)	39 (47.6)	10 (16.1)	47 (27.5)	31 (36.0)	3 (10.0)
	Return to work (full-time);	428 (12.6)	27 (12.3)	11 (13.4)	9 (14.5)	36 (21.1)	19 (22.1)	8 (26.7)
	Return to work (part-time)	340 (10.0)	21 (9.6)	7 (8.5)	7 (11.3)	34 (19.9)	17 (19.8)	10 (33.3)
	Change jobs	245 (7.2)	14 (6.4)	4 (4.9)	3 (4.8)	24 (14.0)	7 (8.1)	3 (10.0)
	Keep receiving disability pensions	288 (8.4)	12 (5.5)	1 (1.2)	8 (12.9)	20 (11.7)	8 (9.3)	5 (16.7)
	Keep retirement benefits/alimony	929 (27.2)	76 (34.7)	20 (24.4)	25 (40.3)	10 (5.8)	4 (4.7)	1 (3.3)
	Expectations – recovery (Yes) [n(%)]	2,031 (59.6)	147 (67.1)	66 (80.5)	39 (62.9)	94 (55.0)	51 (59.3)	20 (66.7)

Functioning & Quality of life	ODI [mean (SD) min-max]	42.7 (16.1) 0-98	43.5 (15.5) 6-90	37.1 (14.2) 6-78	54.0 (13.7) 28-90	45.1 (13.4) 12-88	42.0 (14.7) 12-88	54.3 (9.4) 34-70
	Walking distance [n(%)]							
	<100 m	600 (17.6)	49 (22.4)	15 (18.3)	22 (35.5)	17 (9.9)	6 (7.0)	5 (16.7)
	100-500 m	776 (22.8)	58 (26.5)	19 (23.2)	21 (33.9)	32 (18.7)	12 (14.0)	10 (33.3)
	500 m - 1 km	760 (22.3)	42 (19.2)	16 (19.5)	10 (16.1)	55 (32.2)	28 (17.4)	10 (33.3)
	>1 km	1,274 (37.4)	70 (32.0)	32 (39.0)	9 (14.5)	67 (39.2)	40 (46.5)	5 (16.7)
	Practice sports [n(%)]							
	No	2,130 (62.5)	143 (65.3)	51 (62.2)	47 (75.8)	103 (60.2)	48 (55.8)	17 (56.7)
	Yes top level	28 (0.8)	1 (0.5)	1 (1.2)	0	2 (1.2)	0	0
	Yes recreational level	1,252 (36.7)	75 (34.2)	30 (36.6)	15 (24.2)	66 (38.6)	38 (44.2)	13 (43.3)
	Disability leisure activities (Yes) [n(%)]	3,240 (95.0)	208 (95.0)	75 (91.5)	60 (96.8)	169 (98.8)	84 (97.7)	30 (100)
	SBT item 9 - Bothersomeness (Yes) [n(%)]	2,323 (68.1)	159 (71.7)	51 (62.2)	52 (83.9)	136 (79.5)	63 (73.3)	29 (96.7)
	SF-36 PCS [mean (SD) min-max]	28.9 (8.3) 4-64	28.6 (7.5) 13-50	30.2 (8.3) 14-49	24.8 (6.2) 13-39	28.5 (6.1) 9-43	29.3 (6.5) 12-43	25.5 (5.3) 9-36
	SF-36-MCS [mean (SD) min-max]	45.1 (11.8) 12-74	44.9 (12.2) 15-68	48.2 (12.0) 19-67	42.8 (12.0) 16-62	43.6 (11.6) 21-72	45.5 (11.7) 23-72	38.5 (12.2) 21-65
	SF6D [mean (SD) min-max]	0.576 (0.094) 0.294-0.921	0.572 (0.099) 0.296-0.892	0.598 (0.109) 0.337-0.892	0.535 (0.098) 0.296-0.746	0.561 (0.080) 0.366-0.843	0.578 (0.082) 0.370-0.812	0.516 (0.067) 0.391-0.708
	EQ5D [mean (SD) min-max]	0.447 (0.298) -0.329-1.000	0.444 (0.282) -0.204-0.893	0.511 (0.275) -0.204-0.893	0.304 (0.246) -0.134-0.811	0.405 (0.284) -0.134-0.843	0.448 (0.276) -0.058-0.843	0.266 (0.278) -0.134-0.811
	EQ - VAS [mean (SD) min-max]	56.8 (20.7) 2-100	55.5 (21.1) 4-100	60.1 (20.6) 5-95	47.5 (27.9) 4-90	53.6 (20.9) 4-100	55.0 (20.0) 4-95	47.2 (25.6) 8-100
Outcomes at 1 year follow up	NRS Back pain intensity [mean (SD) min-max]		4.4 (4.1) 0-10	2.4 (2.1) 0-8	6.9 (6.2) 1-10	4.2 (2.4) 0-9	3.3 (2.5) 0-9	6.0 (1.8) 1-8
	NRS Leg pain intensity [mean (SD) min-max]		3.7 (3.1) 0-10	1.7 (2.3) 0-8	5.8 (3.1) 0-10			
	ODI [mean (SD) min-max]		30.4 (18.7) 0-84	11.5 (7.0) 0-22	54.0 (9.9) 42-84	22.7 (17.5) 0-64	8.0 (6.5) 0-22	50.5 (7.0) 42-64
	SF-36 PCS [mean (SD) min-max]		35.9 (10.0) 13-60	45.2 (8.0) 13-60	26.7 (4.3) 13-36	61.4 (20.5) 19-77	70.5 (17.9) 21-97	35.8 (12.7) 19-69
	SF-36-MCS [mean (SD) min-max]		45.9 (10.9) 13-64	52.1 (7.8) 27-64	39.5 (10.5) 19-61	72.8 (18.1) 16-79	80.1 (14.3) 37-99	52.5 (19.4) 16-89
	SF6D [mean (SD) min-max]		0.648 (0.130) 0.662-1.000	0.763 (0.119) 0.528-1.000	0.544 (0.069) 0.338-0.665	0.696 (0.125)0.381-0.948	0.747 (0.125) 0.412-0.948	0.570 (0.075) 0.381-0.795
	EQ5D [mean (SD) min-max]		0.651 (0.278) -0.134-1.000	0.844 (0.098) 0.651-1.000	0.369 (0.300) -0.134-0.811			
	EQ - VAS [mean (SD) min-max]		61.8 (23.0) 1-100	78.9 (17.0) 8-100	43.8 (19.0) 1-90			

CPP Combined Physical and Psychological; *RF* red flag; *SBT* STarT Back Screening Tool (Dutch version)
NRS Numeric Rating Scale; *ODI* Oswestry Disability Index; *SF36-PCS* Short Form 36 – Physical Component Scale; *SF36-MCS* Short Form 36 – Mental Component Scale; *EQ5D* EuroQol 5 Dimensions; *SF6D* Short Form 6 Dimensions
